# French experiences with the online courses on suicidal behaviour, their main features, requests of participants and the opportunities to foster suicide prevention

**DOI:** 10.1192/j.eurpsy.2021.189

**Published:** 2021-08-13

**Authors:** J. Lopez-Castroman, E. Olie

**Affiliations:** 1 Psychiatry, Nimes University Hospital, Nimes, France; 2 Unit 1061, Inserm, Montpellier, France; 3 Psychiatry, University of Montpellier, Montpellier, France; 4 Department Of Emergency Psychiatry & Acute Care, University of Montpellier., Montpellier, France

**Keywords:** self-harm, Education, training program, Teaching

## Abstract

There is a high demand for specific training on the understanding and management of suicidal behaviors. We will present a summary of a massive open online course that was launched in France in 2018 for the first time. The structure of the program was simple: 5 modules presented on a weekly basis, each module contained several short videos with direct training, interviews or discussion. The MOOC offered as well other ressources such as access to a forum during the course, an updated bibliography and vocabulary for each module and an evaluation at the end of the modules. In the first year, the number of registered candidates for the course was very high (>10000) and more than a quarter completed the course (>2500), with participants from 82 countries. The quality of exchanges with the students was very high. The results of this ongoing experience provides interesting insights on how to improve access and motivation to specific training in the field of suicidal behavior for participants from very different backgrounds.

**Disclosure:**

No significant relationships.

